# SIRT1/Nrf2/NF-κB Signaling Mediates Anti-Inflammatory and Anti-Apoptotic Activities of Oleanolic Acid in a Mouse Model of Acute Hepatorenal Damage

**DOI:** 10.3390/medicina59071351

**Published:** 2023-07-24

**Authors:** Manea A. I. Alqrad, Dina S. El-Agamy, Sabrin R. M. Ibrahim, Alaa Sirwi, Hossam M. Abdallah, Essam Abdel-Sattar, Ali M. El-Halawany, Wael M. Elsaed, Gamal A. Mohamed

**Affiliations:** 1 Department of Natural Products and Alternative Medicine, Faculty of Pharmacy, King Abdulaziz University, Jeddah 21589, Saudi Arabia; mibrahimalqrad@stu.kau.edu.sa (M.A.I.A.); asirwi@kau.edu.sa (A.S.); hmafifi@kau.edu.sa (H.M.A.); 2 Department of Pharmacology and Toxicology, Faculty of Pharmacy, Mansoura University, Mansoura 35516, Egypt; dinaagamy@mans.edu.eg; 3 Department of Chemistry, Preparatory Year Program, Batterjee Medical College, Jeddah 21442, Saudi Arabia; sabrin.ibrahim@bmc.edu.sa; 4 Department of Pharmacognosy, Faculty of Pharmacy, Assiut University, Assiut 71526, Egypt; 5 Department of Pharmacognosy, Faculty of Pharmacy, Cairo University, Giza 12613, Egypt; essam.abdelsattar@pharma.cu.edu.eg (E.A.-S.); ali.elhalawany@pharma.cu.edu.eg (A.M.E.-H.); 6 Department of Anatomy and Embryology, Faculty of Medicine, Mansoura University, Mansoura 35516, Egypt; wzaarina@mans.edu.eg

**Keywords:** oleanolic acid, *Viscum schimperi*, thioacetamide, SIRT1/Nrf2/NF-κB, hepatorenal damage, Bax/Bcl-2, health and wellbeing, drug discovery

## Abstract

*Background and objectives:* Oleanolic acid (OA) is a penta-cyclic triterpene with diverse bioactivities such as anticarcinogenic, antiviral, antimicrobial, hepatoprotective, anti-atherosclerotic, hypolipidemic, and gastroprotective. However, its effects on hepatorenal damage remain unclear. The protective activity of OA, separated from *Viscum schimperi* (Loranthaceae), against TAA (thioacetamide)-produced acute hepatic and renal damage was explored. *Materials and Methods:* Mice were treated with OA for 7 days before TAA (200 mg/kg, i.p.). Serum indices of hepatorenal injury, pathological lesions, molecular biological indexes, and inflammatory/apoptotic genes were estimated. *Results:* The tissues of both organs were greatly affected by the TAA injection. That was evident through increased serum markers of hepato-renal injury as well as remarkable histopathological lesions. TAA-induced injury was associated with oxidative and inflammatory responses in both organs as there was an elevation of oxidative stress parameters (4-HNE (4-hydroxy-nonenal), MDA (malondialdehyde), NOx (nitric oxide)), decline of antioxidants (reduced glutathione (GSH), superoxide dismutase (SOD), and total antioxidant capacity (TAC)), and an increase in the gene expression/level of inflammatory mediators (interleukins (1β&6)). The inflammatory response was linked to a significant activation of NF-κB (nuclear-factor kappa-B)/TNF-α (tumor-necrosis factor-alpha) signaling. The inflammatory response in both organs was accompanied by apoptotic changes, including a rise in the gene expression and level of apoptotic parameters (caspase-3 and Bax) along with a decline in Bcl-2 (anti-apoptotic parameter) gene expression and level. These pathogenic events were found to be closely related to the suppression of the antioxidant signaling pathway, Nrf2 (nuclear-factor erythroid 2–related factor-2)/SIRT1 (sirtuin-1)/HO-1 (heme-oxygenase 1). On the other hand, OA significantly ameliorated TAA-induced injury in both organs. On the other hand, OA counterpoised the inflammatory response as it ameliorated NF-κB/TNF-α signaling and cytokine release. OA enhanced Nrf2/SIRT1/HO-1 signaling and counteracted apoptotic damage. *Conclusions:* OA showed anti-inflammation and antiapoptotic capacities that effectively suppressed TAA-induced acute hepatorenal damage.

## 1. Introduction

Many toxins have been used to establish appropriate animal models of acute multi-organs toxicity for exploring novel potential anti-inflammatory therapeutics. The pathological lesions caused by these toxins are like many kinds of inflammatory dysfunction. One of these toxicants is thioacetamide (TAA), which is an organosulfur compound used in many industries [1,2,3]. TAA causes toxic injuries in multiple organs such as the brain, kidney, intestine, liver, lung, spleen, and stomach, resulting in functional and structural abnormalities ranging from necrosis, and fibrosis to cirrhosis [4]. The deleterious effect of TAA depends on its dose and the exposure time; i.e., a single-dose administration produces hepatic injury in the form of apoptosis, centrilobular hepatic necrosis, and peri-portal inflammatory cell infiltration, while long-time exposure leads to liver cirrhosis and bile duct proliferation [4,5,6].

Many pathogenic mechanisms interplay during TAA-induced injuries, including oxidative stress, inflammation, and apoptosis. TAA produces a state of oxidative stress because of the formation of reactive unstable metabolites (TAA-S-oxide and TAA-S-dioxide), which enhance the ROS (reactive oxygen species) generation that promotes free radical-mediated damage to cellular components (e.g., DNA, proteins, and lipids) [1,7]. Additionally, the TAA metabolites covalent binding to antioxidant enzymes results in a repressed cellular antioxidant capacity in the liver and kidney and eventually leads to their injuries within 6 to 12 h after TAA incorporation [4,8]. Furthermore, TAA produces inflammation due to the augmentation of the expression of inflammatory cytokines such as IL-1β (interleukin-1β) and TNF-α (tumor-necrosis factor-alpha) [3]. TAA-induced oxidative stress and inflammation lead to systematic apoptosis within the kidney and liver via modulation of apoptotic/antiapoptotic genes [9].

Medicinal plants play a prime role in treating renal and hepatic disorders due to their phytoconstituents that possess hepato- and nephroprotective capacities through various mechanisms [10,11,12,13]. These phytochemicals belong to various chemical classes, including phenylpropanoids, xanthones, phenolic acids, terpenoids, lignans, stilbenoids, curcuminoids, carotenoids, alkaloids, saponins, and flavonoids [10,11,12,13,14,15,16,17,18].

The *Viscum* genus (Loranthaceae) comprises 100 species that are commonly found in tropical and temperate areas of Africa, Europe, Australia, and Asia [19]. These species have attracted remarkable interest considering their extensive applications in food resources, clinical therapy, and healthcare products worldwide [19,20]. They are employed as a remedy for multiple disorders such as tumors, fetal restlessness, inflammation, arthritis, hypertension, hepatitis, constipation, rheumatism, stomach ulcers, internal hemorrhages, and neurodegenerative, blood, inflammatory, and skin diseases [19]. Peptides, alkaloids, terpenoids, phenolics, viscotoxins, and flavonoids are the main constituents reported from this genus [19,21,22].

*V. schimperi* Engl. is among the least studied species of this genus that is found in Saudi Arabia [22,23,24]. Its phytochemical study revealed the existence of phenolic acids (e.g., quinic, caffeic, sinapic, ferulic, and rosmarinic acids), flavonoids (e.g., viscutin, luteoliflavan glycoside, erodictyol, erodictyol glycoside, and isorhamnetin glucoside), and triterpenes (e.g., betulinic and oleanolic acids) [23,24,25]. This plant demonstrated hypolipidemic and antidiabetic activities [23,24]. Oleanolic acid (OA) is widely used in Chinese medicine for treating inflammatory disorders. OA showed protective activities against injurious agents such as lipopolysaccharide/D-galactosamine, acetaminophen, and cisplatin. However, its effect against thioacetamide (TAA)-induced acute hepatic and renal injury has not yet been tested [26,27,28]. In this context, OA was separated from *V. schimperi* and its beneficial effects were assessed against TAA-induced acute hepatorenal damage. Furthermore, the possible mechanistic pathways associated with its protective activities were investigated.

## 2. Materials and Methods

### 2.1. General Procedures

A DRX 850 MHz Bruker Avance spectrometer was employed for NMR spectra recording. TLC and column chromatography were carried out using SiO_2_ 60 F254 TLC plates and a SiO_2_ 60 column, respectively. The purity of the compound was checked using HPLC [Zorbax column (250 × 4.6 mm, acetonitrile:H_2_O (50:50); flow rate: 1 mL/min] equipped with a BIP-I HPLC JASCO pump and a RI-8020 TOSOH detector (see Supplementary Materials).

### 2.2. Plant Materials

*Viscum schemperi* Engl. was gathered in March 2019 from Al-Taif governorate/Saudi Arabia. The plant’s verification was authenticated by Dr. Emad AlSharif (Faculty of Science and Arts/King Abdulaziz University). A specimen (No. VS1167A) was deposited in the Department of Natural Products and Alternative Medicine herbarium.

### 2.3. Extraction and Isolation of OA

**Figure 1:**
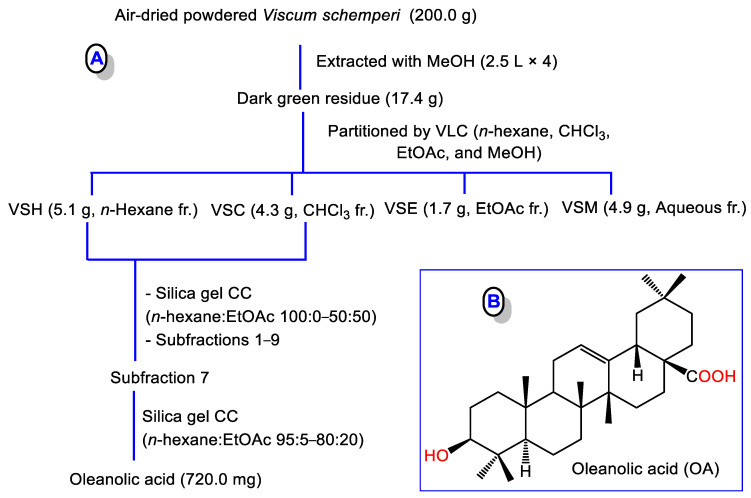
(**A**) Scheme for isolation of oleanolic acid (OA); (**B**) Chemical structure of OA.

The powdered *V. schemperi* (200.0 g) was extracted with MeOH (2.5 L × 4). The concentrated combined extract (dark green residue, 17.4 g) was subjected to chromatographic separation to afford OA (Figure 1).

### 2.4. Materials and Chemicals

Lipid peroxidative parameters MDA (malondialdehyde) and 4-HNE (4-Hydroxynonenal) were purchased from Abcam (ab233471, Cambridge, UK) and MyBiosource (MBS027502/USA), respectively. TAA was supplied by Sigma Aldrich. Antioxidant status parameters, SOD (superoxide-dismutase), GSH (reduced-glutathione), and TAC (total-antioxidant capacity), were obtained from Calbiochem/MERCK (354102,100T Millipore, Darmstadt, Germany), Abcam (ab65354, Cambridge, UK), and (MAK187.1KT, Sigma-Aldrich St. Louis, MO, USA), respectively. A NOx (nitric-oxide) kit was supplied by Bio-Diagnostic Co. (Giza, Egypt). ELISA kits for the NF-κB/inflammatory cytokines (IL-6, TNF-α, IL-1β), heme-oxygenase (HO-1), and apoptotic (caspase-3 and Bax)/anti-apoptosis (Bcl-2) markers were procured from Cusabio_Biotech CO (Shanghai, China). Nuclear extract, TransAM NF-κB p65, and TransAM Nrf2 binding activity kits were purchased from Active_Motif Inc. (Carlsbad, CA, USA).

### 2.5. Animals

Under typical circumstances of a 12-h light/dark cycle, humidity, and temperature, male BALB/c mice (≈25 g, 6–8 weeks age) were maintained for the duration of the experiments. The work protocol (RES-2022-0064) was accepted by the Batterjee_Medical_College’s Research Ethical Committee, which closely aligns with the NIH guidelines for experimental animals’ usage and care.

### 2.6. Experimental Procedures

Acute hepatic and kidney injury was induced by a single 200 mg/kg TAA intraperitoneal injection as previously described [29]. Mice (n = 30) were randomly divided into five sets (6 mice/group): The control (CTRL) group, where mice received normal saline; the OA group that was treated with OA (90 mg/kg/day for 7 days); the TAA group, where mice were injected with TAA; the OA 45 + TAA and OA 90 + TAA groups, where mice received OA at 45 and 90 mg/kg, respectively, for 7 days before TAA challenge.

24 h after TAA administration, mice were humanely sacrificed under anesthesia with xylazine and ketamine (10 and 75 mg/kg, respectively). From each animal, the collected blood was centrifuged to obtain the serum, which was kept at −80 °C for analysis. Parts of the liver and kidney were harvested for biological, histological, immunohistochemical (IHC), ELISA, and RT-PCR analyses.

#### 2.6.1. Serum Markers of Hepatorenal Damage

ALT and AST (Aminotransferases) and LDH (lactate- dehydrogenase) in serum were measured according to the kits’ protocol (Human, Wiesbaden, Germany). In brief, the samples were mixed with the working reagent (for ALT: L-alanine, TRIS buffer, 2-oxoglutarate, and NADH; for AST: L-aspartate, TRIS buffer, 2-oxoglutarate, and NADH; for LDH: TRIS buffer, pyruvate, and NADH). The absorbance’s change/min (340 nm) was recorded for 3 min using a spectrophotometer (UNICO Instruments C., Model 1200, NJ, USA). For ALP, samples were mixed with diethanolamine buffer/magnesium chloride and p-nitrophenyl phosphate. The absorbance change/min (405 nm) was recorded for 3 min. BUN (blood-urea-nitrogen) and creatinine were determined in serum. For creatinine, the samples were mixed with the working reagent (picric acid and sodium hydroxide). After 30 s, the absorbance of the sample/standard was read (492 nm) and 2 min later the absorbance of the sample/standard was read. For BUN, the samples were mixed with the working reagent (Tris-Buffer; Uurease; GLDH; NADH; adenosine-5-diphosphate; α-oxoglutarate) at 37 °C, then the absorbance’s change/min (340 nm) was recorded for 3 min.

#### 2.6.2. Oxidative and Antioxidant Markers

In ice-cold buffer (50 mM potassium phosphate, 1 mM EDTA, PH 7.5), a piece (≈50–100 mg) of the hepatic and the kidney tissues was separately homogenized and centrifuged (10 min/4 °C/3000× *g*) to obtain the supernatants, which were kept at 4 °C for further assays using commercial kits as follows:

##### MDA

MDA assessment relies on the production of a colored product by a reaction with thiobarbituric acid that was colorimetrically measured employing a T80+ UV/VIS spectrophotometer at 532 nm (PG Instruments Ltd., Lutterworth, UK).

##### 4-HNE

HRP-conjugate reagent was added to samples, covered with a closure-plate membrane, and then incubated at 37 °C for 60 min before washing four times. Finally, at 37 °C away from light, the chromogen solution was added for 15 min before adding the stop solution and measuring optical density at 450 nm.

##### GSH

Its assay is based on the reaction between all mercaptans (RSH) in the samples and 4-chloro-1-methyl-7-trifluromethyl-quinoliniumm-ethylsulfate and then β-elimination reaction under basic condition (30% NaOH), which specifically changes the substitution product obtained with GSH into a chromophoric thione with absorbance maxima at 400 nm.

##### SOD

The assay depends on the formation of formazan dye after mixing the samples, enzyme working solution, and WST-1 and incubation at 37 °C for 20 min. The absorbance was read at 450 nm.

##### TAC

Samples were mixed with a Cu^2+^ working solution and incubated for 90 min at 25 °C. The absorbance was measured at 570 nm.

##### NO**x**

The tissue was homogenized using an ice-cold buffer (100 mM potassium phosphate, pH 7.0) containing 2 mM EDTA and centrifuged (10 min/4 °C/4000× *g*). N-(1–naphthyl)ethylenediamine and sulphanilamide were added to samples to form a reddish-purple product that was spectrophotometrically quantified at 540 nm.

#### 2.6.3. Histology and Immunohistochemistry (IHC)

For hematoxylin and eosin (H&E) stain, liver and kidney tissues were fixed in neutral buffered formalin, ethanol dehydrated, and paraffin embedded. Paraffin blocks were cut and stained with H&E. Sections were randomly examined in a blind manner and the pathological injuries were graded as previously described [30,31,32].

For IHC, paraffin sections of the liver and kidney were dewaxed and handled as previously demonstrated [30,33]. IHC was carried out using the primary antibodies: Nrf2 (1:200) (Fisher Scientific Inc., Waltham, MA, USA), rabbit-polyclonal-antibody against NF-κB p65 (1:200), caspase-3 (1:200), and Bcl2 (1:200) (Elabscience Biotechnology Inc., Houston, TX, USA). Diaminobenzidine (DAB) was utilized for visualization.

#### 2.6.4. ELISA

A piece of hepatic/kidney tissue was homogenized in PBS and centrifuged to obtain the supernatants for the estimation of the protein level of NF-κB/inflammatory cascade (TNF-α, IL-1β, IL-6), HO-1 (heme-oxygenase), and apoptosis/anti-apoptosis markers (Bax, Bcl-2, and caspase-3) according to the ELISA kits provided protocol. NF-κB p65 activation and Nrf2 binding activity were estimated in the nuclear extract as described in the kit’s guidelines. The nuclear extract of the tissue was obtained based on the protocol of the kit. In brief, a small piece of the liver or kidney tissue was weighed, crushed, and mixed with hypotonic buffer containing phosphatase and proteases inhibitors, as well as dithiothreitol (DTT) until single cell slurry was obtained, which is kept on ice for 15 min. After centrifugation (4 °C/10 min), the cell pellets were resuspended in hypotonic buffer, centrifuged, mixed with detergent, and centrifuged (4 °C/30 s). The nuclear pellets were resuspended in a lysis buffer containing DTT and a protease inhibitor cocktail. After rocking on ice for 30 min, the samples were centrifuged to obtain the nuclear extract. The protein content of the nuclear extract was measured before the estimation of NF-κB p65 activation and Nrf2 binding activity using an ELISA kit. Results were represented as OD 450 nm.

#### 2.6.5. RT-PCR

The expressions of IL-1β, IL-6, TNF-α, Bax, Bcl2, caspase-3, SIRT1, Nrf2, and HO-1 were determined using RT-PCR. In brief, RNA was obtained using the QIAzol reagent (Qiagen, Hilden, Germany) according to the kit’s instructions. The RNA concentration was estimated using the NanoDrop 2000 (ThermoScientific, Waltham, MA, USA). Reverse transcription of RNA samples (≈1 µg) was performed using the Bioline cDNA synthesis kit (Bioline, Taunton, MA, USA). cDNA replication was carried out using RT-PCR equipment (Pikoreal 96, ThermoScientific, USA). The amplification process was composed of a total volume mixture (20 µL): HERA SYBR green PCR Master Mix (10 µL, Willowfort, West Midlands, UK), cDNA template (2 µL), 2 µL of each gene primer (10 pmol/µL), and nuclease-free water (6 µL). The process was carried out according to the following conditions: 95 °C for 2 min, followed by 40 cycles of 95 °C for 10 s, and 60 °C for 30 s. The studied genes primers were designed utilizing Primer3Plus software, and their specificity was assigned utilizing the Primer-BLAST program. Primer sets synthesis was carried out using Vivantis. GAPDH (Glyceraldehyde-3-phosphate dehydrogenase) was used as a control gene (Table 1). Relative gene expression levels were represented as ∆Ct = Ct target gene − Ct control gene; the fold change in gene expression was calculated according to the 2^−∆∆CT^ method.

### 2.7. Data Analysis

Data (mean ± SEM) were compared using a one-way analysis of variance (one-way ANOVA) and the Tukey-Kramer multiple comparisons test as a post hoc test. A *p*-value of <0.05 was considered a significant difference.

## 3. Results

### 3.1. Purification and Identification of OA

The MeOH extract of *V. schemperi* was chromatographed using VLC (vacuum liquid chromatography). The combined *n*-hexane and CHCl_3_ fractions were separated on SiO_2_ CC to afford OA, which was specified by comparing its spectral data with the literature (Figures S1–S4 and Table S1) [34].

### 3.2. Functional Status and Histopathology of Liver and Kidney

**Figure 2:**
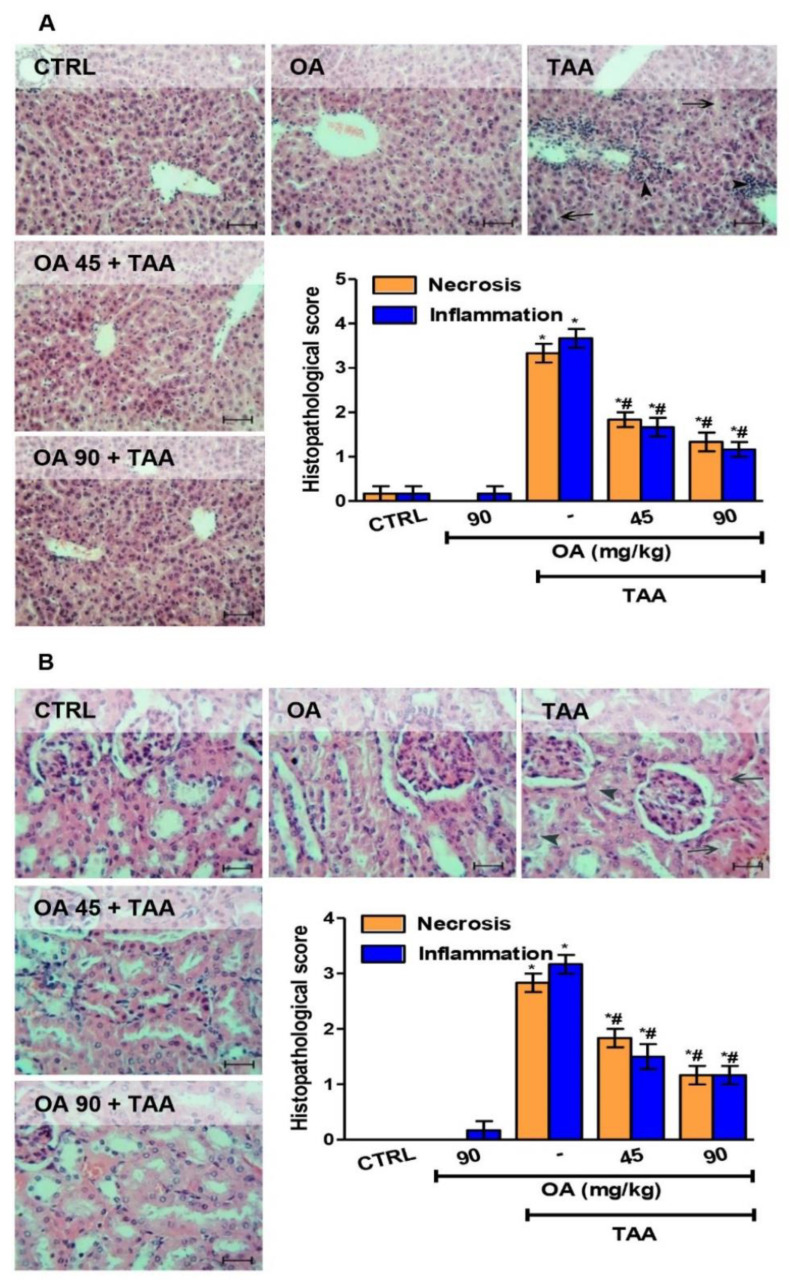
Oleanolic acid (OA) mitigated thioacetamide-induced acute hepatorenal damage. (**A**) Liver sections show the normal liver structure of the control and OA groups, while the TAA group showed disruption of the normal liver architecture with obliteration of the interhepatic sinusoids (arrows) and diffuse inflammatory cell infiltration (arrow heads) in the vicinity of the central veins. The OA + TAA groups exhibited less marked pathological changes in variable degrees (hematoxylin and eosin × 200). (**B**) Kidney sections showing normal the structure of the control and OA groups. The TAA group exhibited cytoplasm vacculation (arrow heads) and congestion (arrows). The OA + TAA groups showed remarkable improvement in TAA-induced changes (hematoxylin and eosin × 200). Data are the mean ± SE (n = 6). * *p* < 0.05 vs. control group; ^#^ *p* < 0.05 vs. TAA group (one-way ANOVA).

TAA administration altered the functional state of the liver and kidney. As shown in Table 2, TAA produced significant rises in serum markers of hepatic and kidney injury (ALT, AST, ALP, LDH, BUN, and creatinine) compared to normal animals. Histopathological examination revealed lesions in both organs in the form of necrosis, congestion, inflammatory cell infiltration, and apoptotic changes, as shown in Figure 2.

OA pre-treatment significantly protected the liver and kidney as it ameliorated the abovementioned serum parameters of injury and improved the pathological changes observed in both organs (Figure 2).

### 3.3. Antioxidants and Oxidative Stress in the Kidney and Liver

As shown in Table 3, TAA injection resulted in an elevation of lipid peroxidative markers; MDA and 4-HNE in both organs. In addition, NOx (a marker of nitro-oxidative damage) was significantly increased. Furthermore, there was a significant decrease in antioxidants (GSH, SOD, and TAC) in the hepatic and kidney tissues. OA pre-treatment opposed lipid peroxidation and enhanced the antioxidant status of both organs.

### 3.4. Inflammatory Response in the Liver and Kidney

**Figure 3:**
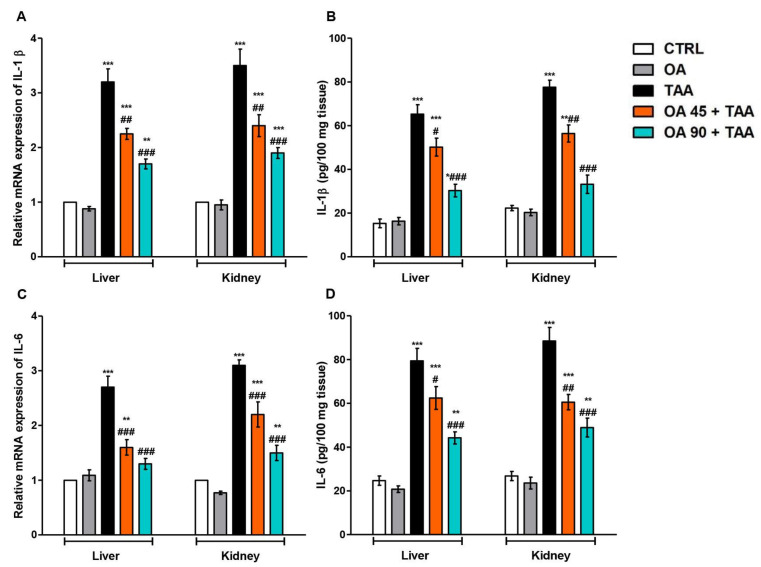
Oleanolic acid (OA) lessened thioacetamide (TAA)-induced upregulation and release of interleukins (IL-1β&6). (**A**,**B**) Relative mRNA expression and level of IL-1β; (**C**,**D**) Relative mRNA expression and level of IL-6. Data are the mean ± SE (n = 6). * *p* < 0.05, ** *p* < 0.01, *** *p* < 0.001 vs. control group; ^#^ *p* < 0.05, ^##^ *p* < 0.01, ^###^ *p* < 0.001 vs. TAA group (one-way ANOVA).

Inflammatory cytokines, IL-1β and IL-6, were measured to evaluate the TAA-induced inflammatory response in the liver and kidney. Significant elevations in both ILs were observed in the liver and kidney of the TAA group compared to the control group. OA supplementation reversed the elevation of these inflammatory markers in both organs compared to the TAA group (Figure 3).

### 3.5. NF-κB/TNF-α Inflammatory Signaling Pathway in the Kidney and Liver

**Figure 4:**
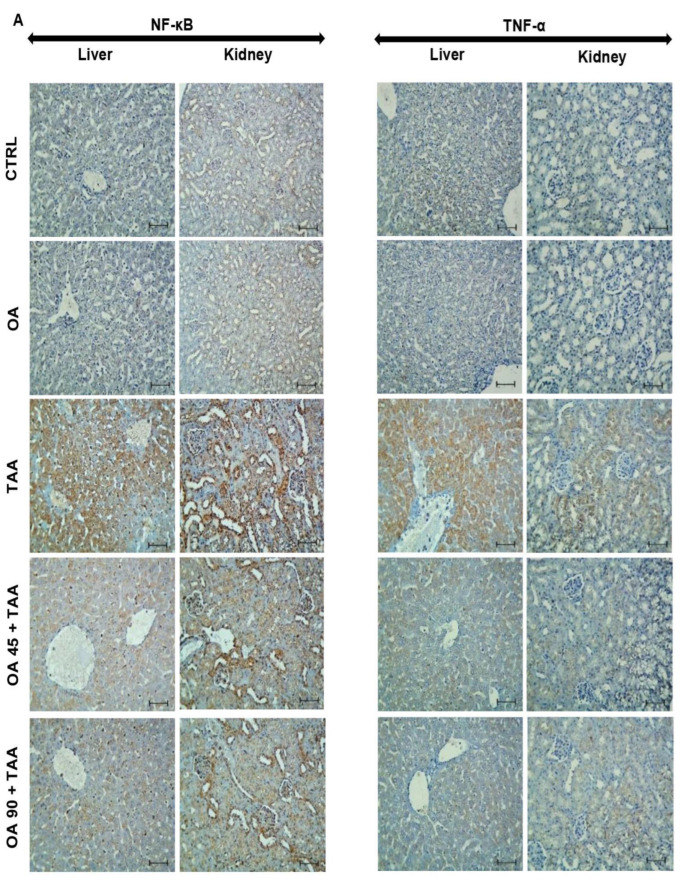

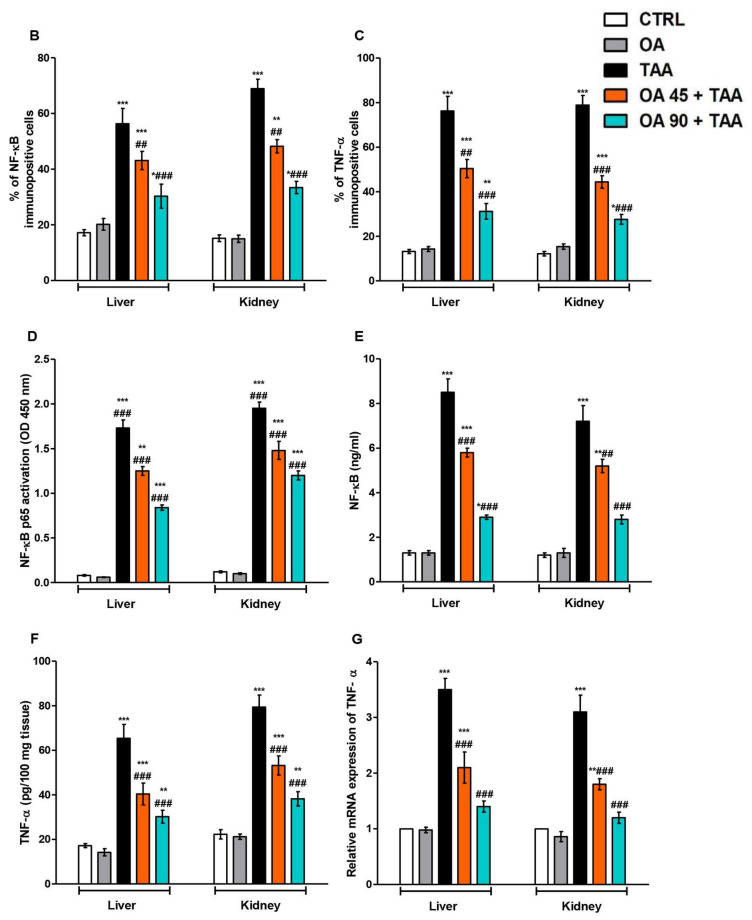
Oleanolic acid (OA) attenuated thioacetamide (TAA)-induced activation of NF-κB/TNF-α signalling. (**A**) Immunostaining of NF-κB and TNF-α of the liver and kidney tissue; (**B**,**C**) % of immuno-positive cells of NF-κB and TNF-α respectively; (**D**,**E**) Activation and level of NF-κB respectively; (**F**,**G**) Level and mRNA of TNF-α. Data are the mean ± SE (n = 6). ^##^ *p* < 0.01, ^###^ *p*< 0.001 vs. TAA group * *p* < 0.05, ** *p* < 0.01, *** *p* < 0.001 vs. control group (one-way ANOVA).

As presented in Figure 4, TAA enhanced NF-κB p65 activation, the levels, protein immuno-expression of NF-κB/TNF-α, and mRNA expression of TNF-α in the hepatic and kidney tissues. OA pre-treatment suppressed these changes, and hence, antagonized the TAA-induced activation of NF-κB/TNF-α signaling in both organs.

### 3.6. Apoptotic Changes in the Liver and Kidney

**Figure 5:**
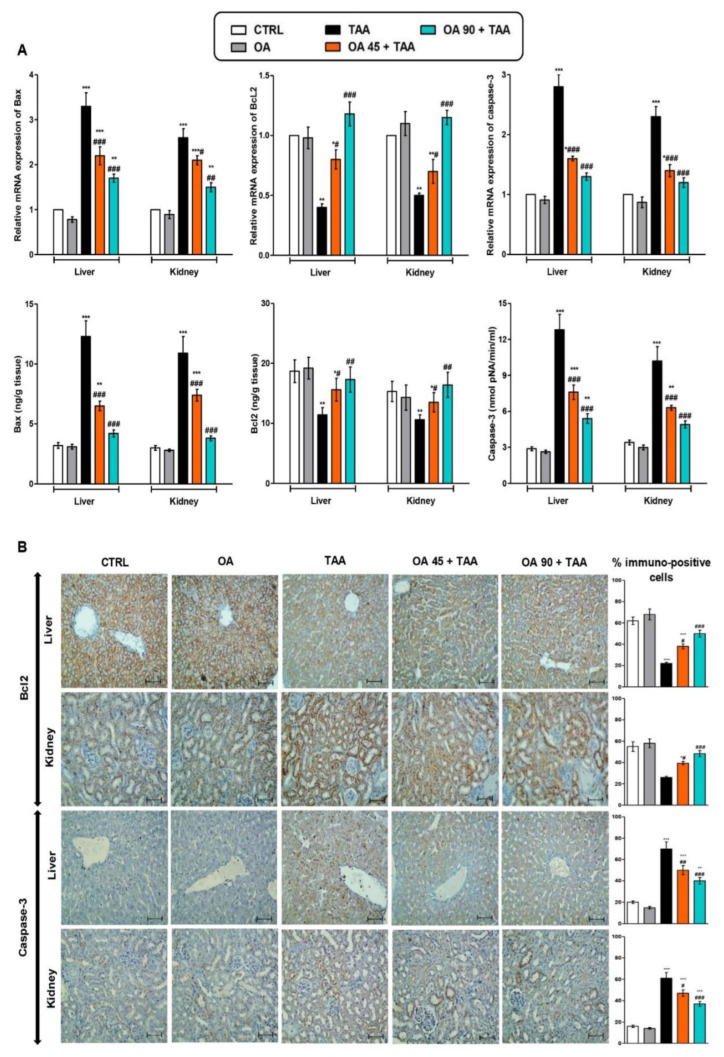
Oleanolic acid (OA) offset thioacetamide (TAA)-induced apoptosis in liver and kidney. (**A**) mRNA and level of Bax, Bcl2 and caspase-3; (**B**) Immunostaining of Bcl2 and caspase-3 of the hepatic and kidney tissue. Data are the mean ± SE (n = 6). * *p* < 0.05, ** *p* < 0.01, *** *p* < 0.001 vs. control group; ^#^ *p* < 0.05, ^##^ *p* < 0.01, ^###^ *p* < 0.001 vs. TAA group (one-way ANOVA).

TAA administration provoked the elevation of the genetic expression and protein level of apoptosis markers (Bax and caspase-3) and decreased the level and genetic/protein immuno-expression of Bcl2. Contrarily, OA pre-treatment antagonized TAA-induced apoptotic changes and almost normalized the genetic expression and protein levels of the abovementioned parameters (Figure 5).

### 3.7. SIRT1/Nrf2/HO-1 Signaling in Liver and Kidney

**Figure 6:**
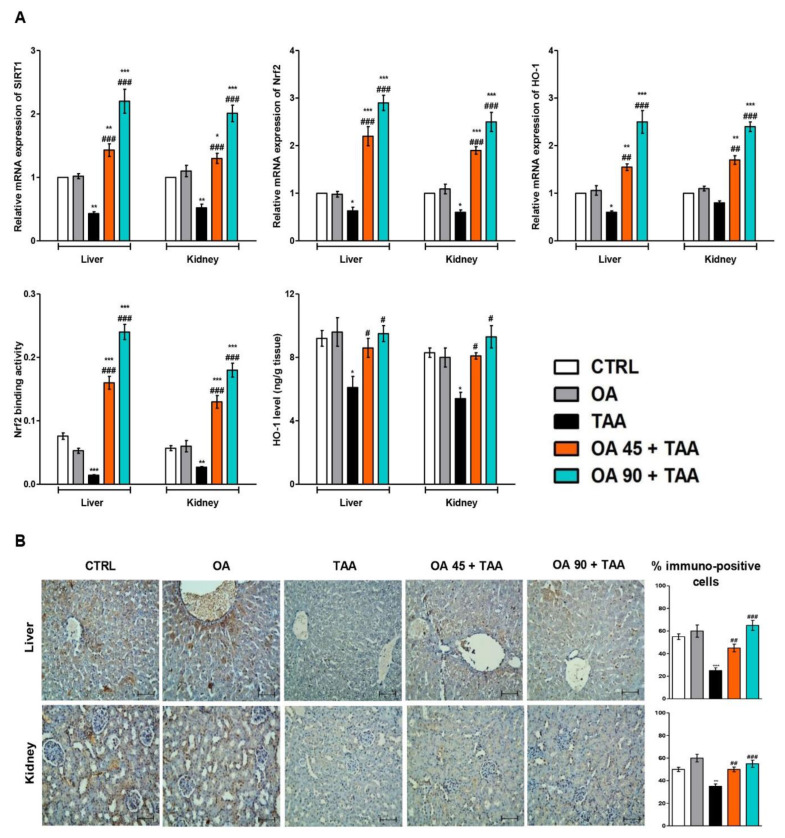
Oleanolic acid (OA) augmented SIRT1/Nrf2/HO-1 signaling in the liver and kidney. (**A**) mRNA expression of Nrf2/SIRT1/HO-1; Nrf2 binding activity, and HO-1 level. (**B**) Nrf2 immuno-expression in the kidney and liver tissue. The TAA specimen possessed minimal immuno-stain compared to the control group while the OA-pre-treated groups showed much higher Nrf2 immuno-stain. Data are the mean ± SE (n = 6). * *p* < 0.05, ** *p* < 0.01, *** *p* < 0.001 vs. control group; ^#^ *p* < 0.05, ^##^ *p* < 0.01, ^###^ *p* < 0.001 vs. TAA group (one-way ANOVA).

As presented in Figure 6, TAA injection resulted in the repression of SIRT1/Nrf2/HO-1 signaling as there was significant downregulation in the gene expression of SIRT1/Nrf2/HO-1 as well as the protein immuno-expression of Nrf2. Furthermore, Nrf2 binding activity was repressed, and the HO-1 level was significantly reduced compared to the control animals. The OA pre-treatment counteracted these changes and boosted the expression and levels of SIRT1/Nrf2/HO-1 compared to the TAA group.

## 4. Discussion

TAA is a powerful hepato- and nephrotoxic agent that is widely used to establish a reproducible model of acute liver and kidney injury [2,3,4,5,6,7,8,9]. OA is a penta-cyclic triterpene that exists in free or glycosidic forms with one or more sugar moieties. OA is a component of various plant foods (e.g., pears, apples, tomatoes, grapes, olives, ginger, strawberries, and mango), medicinal and culinary herbs (e.g., oregano, rosemary, sage, fennel, basil, olive leaf, and ginseng), and thus, it is an integral part of the human diet [35]. The present study illustrated the anti-inflammatory and anti-apoptotic capabilities of OA to mitigate TAA-induced hepatorenal damage via a modulating of the SIRT1/Nrf2/NF-κB/inflammatory cytokines cascade that suggests the potential beneficial protective activity of OA against acute hepatorenal damage.

Serum transaminases (AST and ALT), ALP, and LDH reflect the cellular integrity of hepatic tissues as their high levels indicate a membrane permeability alteration and eventual enzymes leakage into the blood [36]. Creatinine concentration is a substantial marker of renal function. The increase in serum creatinine indicates leakage from necrotic cells or its biosynthesis upregulation. BUN reflects the accumulated urea amount that is not effectively excreted due to kidney impairment [5,37]. In this study, TAA injection affected hepatic and kidney function as presented by elevated levels of serum transaminases, creatinine, and BUN. Alongside, TAA-induced deteriorated histopathology of the liver and kidney. These data agree with the former reports that demonstrated the injurious effects of TAA on the kidney and liver [38,39,40]. Contrarily, OA-pretreated mice showed significant restoration of elevated levels of serum markers of hepatic and renal toxicities, as well as improvement in the histopathological analysis of the liver and kidney. These data demonstrated the protective activity of OA against TAA-induced liver and kidney injury. These results were in harmony with the previously documented hepato-and reno-protective activities of OA against other experimental models resulting from different injurious agents [26,27,28]. These promising results led us to investigate the possible mechanisms that are responsible for OA protective activities.

TAA-induced toxicity is strongly correlated with exacerbated lipid peroxidation and oxidative stress due to the formation of *S*-oxide and *S*-dioxide metabolites, which are highly reactive electrophilic compounds that attack and bind to various cellular macromolecules, leading to lipid peroxidative damage [2,36,41,42]. Furthermore, indiscriminate cellular destruction by an elevated level of NOx and its reactive nitrogen species had been reported in TAA-induced hepatotoxic damage [43]. Our results emphasized the existence of oxidative damage due to TAA administration, as there was a significant increase in lipid peroxidative markers (MDA and 4-HNE) and a nitro-oxidative mediator (NOx), which was accompanied by diminished antioxidant status (GSH, SOD, and TAC) in liver and kidney tissues. Notably, OA treatment potentially augmented the antioxidants concurrently with a reduction in lipid peroxidation products. In this context, our data suggested that OA could safeguard cells from oxidative damage via the enhancement of antioxidant enzymes and the ensuing suppression of lipid peroxidation. These results are in line with previous reports that demonstrated the antioxidant effects of OA against other models of oxidative damage [44,45,46].

Oxidative stress is a crucial promotor for inflammatory signaling in TAA-induced hepatic and kidney lesions. The sustained state of lipid peroxidative damage and impairment of the antioxidant defense system led to an aggravation of hepatic inflammation and necrosis, with a subsequent release of pro-inflammatory cytokines as interleukins [44]. Our data demonstrated the enhancement of the genetic expression and release of IL-1β and IL-6, which confirmed the incidence of oxidative-inflammatory reactions in the liver and kidney following the TAA challenge.

NF-κB is considered a cornerstone in the mediation of the inflammatory response following TAA injection [47]. The activation of NF-κB results in a significant enhancement of the genetic expression and overproduction of inflammatory cytokines, such as TNF-α and ILs. TNF-α is crucial for TAA-induced inflammation as it regulates the release of other proinflammatory mediators, such as IL-1β and NO, that complete the inflammatory response [48,49,50]. Our data are in the same line with the previous reports, as there was a significant enhancement of NF-κB p65 activation, as well as an increase in the level/genetic expression and protein immuno-expression of NF-κB/TNF-α in the TAA group. Conversely, OA pre-treated animals showed remarkable suppression of NF-κB p65 activation and a lowering in the level and expression of NF-κB/TNF-α, suggesting the ability of OA to modulate NF-κB/TNF-α signaling. Interestingly, OA was previously reported to inhibit NF-κB activation in other experimental inflammatory models [51,52,53]. These inhibitory effects of OA on NF-κB/TNF-α signaling may be responsible in part for the anti-inflammatory effects of OA and its hepatoprotection activities against TAA-induced injury in the liver and kidney.

In addition to oxidative and inflammatory responses, apoptotic changes participate in the mediation of TAA-induced injury to a great extent. Apoptosis results from the imbalance between the pro-apoptotic and anti-apoptotic proteins, which usually arise from the upregulation of the pro-apoptotic proteins and the downregulation of the anti-apoptotic ones [54]. Activating apoptotic proteins such as Bax and its translocation into the mitochondria result in a Bcl-2 family imbalance, which then mediates cell apoptosis and mitochondrial dysfunction. The apoptotic molecules expression is driven in part by NF-κB signaling and oxidative stress, as NF-κB upregulates the expression levels of apoptotic genes of the Bcl-2 family [55]. Our results showed the implication of apoptotic changes during TAA-induced hepatic and kidney damage. There was a significant increase in the genetic expression/levels of Bax and caspase-3 (pivotal apoptotic mediators). Additionally, genetic expression/levels of Bcl-2 were significantly decreased. Notably, OA reversed these apoptotic changes, promoted Bcl-2 expression, and inhibited Bax/caspase-3 expression. These data are aligned with the previously reported anti-apoptotic effects of OA against apoptosis in experimental subarachnoid hemorrhage [44].

SIRT1, a member of the Sirtuin family, is an epigenetic regulator that has been reported to be vital in the modulation of physiological and cellular processes in pathological and normal states. SIRT1 exhibited an inverse link to NF-κB in inflammation regulation and is a key regulator of the inflammatory responsiveness in the liver [50]. Reduced SIRT1 expression exacerbates the inflammatory response via NF-κB transcription and increases the reactive oxygen species formation [56]. Our findings indicated the implication of SIRT1 downregulation in the pathogenic events following TAA injection, which agreed with the previous studies [57]. OA enhanced the expression of SIRT1, which is in line with the previous investigations [44].

Nrf2 binds to AREs (antioxidant response elements) in the promoter region of a number of genes encoding antioxidant enzymes. Normally, Nrf2 is present in its inactive state in the cytoplasm, repressed by Keap1 (Kelch-like ECH-associated-protein-1). Nrf2 becomes activated via many stimuli, including oxidative stress, where it is released from its repressor and translocated into the nucleus, where it stimulates the expression of many antioxidants. Nrf2 activators are known to protect against oxidative damage [58]. Our results indicated the suppression of Nrf2/HO-1 signaling after TAA injection. These data are supported by former studies that demonstrated the important role of the Nrf2 pathway in the pathogenesis of TAA-induced injury [59,60,61]. Importantly, OA pre-treatment showed a potent enhancement of the Nrf2/HO-1 pathway, which may be partially accountable for the protective action against TAA-induced hepatorenal injury. Notably, many previous reports had attributed the protective potential of OA to its ability to activate Nrf2 [44,58,62].

## 5. Conclusions

**Figure 7:**
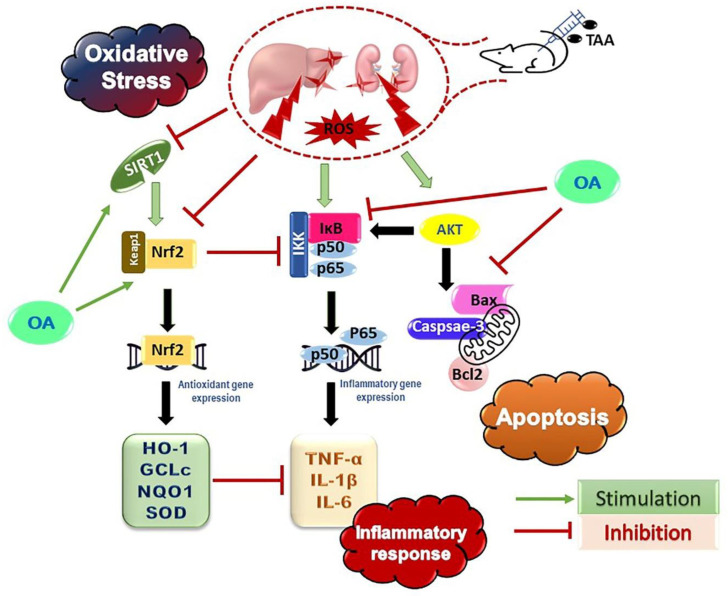
Summary of the molecular pathways modulated by oleanolic acid (OA) against thioacetamide (TAA)-induced acute hepatic and kidney injury.

In summary, this study revealed the efficacy of OA to counteract TAA-induced hepatic and kidney injury in mice through the reduction of hepatocyte oxidative damage, the suppression of inflammation, and apoptosis. More importantly, OA repressed TAA-produced hepatic and kidney injury by inhibiting NF-κB/TNF-α-mediated inflammation/apoptosis and enhancing the SIRT1/Nrf2/HO-1 signaling pathway (Figure 7). These promising pharmacological activities suggest the potential use of OA against hepatorenal damage.

## Figures and Tables

**Table 1:** RT-PCR Primers’ sequences.

Gene (Mouse)	Sequence (5′-3′)	Product (bp)
*IL-1β*	F: GCAACTGTTCCTGAACTCAACT	81
	R: GGGTCCGTCAACTTCAAAGA	
*IL-6*	F: AGTCCTTCCTACCCCAATTTCC	79
	R: GGTCTTGGTCCTTAGCCACT	
*TNF-α*	F: TGAACTTCGGGGTGATCGGT	99
	R: GGTGGTTTGTGAGTGTGAGGG	
*Bax*	F: TGAAGACAGGGGCCTTTTTG	140
	R: AATTCGCCGGAGACACTCG	
*Bcl2*	F: CCTGTGGATGACTGAGTACCTG	123
	R: AGCCAGGAGAAATCAAACAGAGG	
*Caspase-3*	F: ATGGAGAACAACAAAACCTCAGT	74
	R: TTGCTCCCATGTATGGTCTTTAC	
*SIRT1*	F: CGATGACAGAACGTCACACG	111
	R: ATTGTTCGAGGATCGGTGCC	
*Nrf2*	F: AAGAATAAAGTCGCCGCCCA	170
	R: AGATACAAGGTGCTGAGCCG	
*HO-1*	F: GAAATCATCCCTTGCACGCC	122
	R: CCTGAGAGGTCACCCAGGTA	
*Glyceraldehyde-3-phosphate dehydrogenase (GAPDH)*	F: AGGTCGGTGTGAACGGATTTG	123
	R: TGTAGACCATGTAGTTGAGGTCA	

**Table 2:** Oleanolic acid (OA) attenuated serum parameters of thioacetamide-induced acute hepatorenal damage.

Serum Parameters	Groups				
	Control	OA	TAA	OA 45 + TAA	OA 90 + TAA
ALT (IU/L)	38 ± 4.8	33.6 ± 3.3	456 ± 26.1 *	290 ± 25 *^##^	159.3 ± 8.3 *^##^
AST (IU/L)	49.2 ± 3.8	51 ± 6	699 ± 33.1 *	419.8 ± 19.4 *^##^	260 ± 27.6 *^##^
ALP (IU/L)	37.2 ± 2.9	33.4 ± 3.1	535.7 ± 51.6 *	336 ± 40.3 *^##^	252.8 ± 25.1 *^##^
LDH (IU/L)	65.1 ± 10.6	73.8 ± 9.7	945.8 ± 70.5 *	500.8 ± 44.7 *^##^	350.5 ± 23.9 *^##^
BUN (mg/dL)	20.7 ± 1.5	24.2 ± 1.9	48.6 ± 3 *	37.8 ± 3.3 *^#^	28.2 ± 2.4 *^##^
Creatinine (mg/dL)	0.9 ± 0.07	0.9 ± 0.07	2.5 ± 0.1 *	1.8 ± 0.1 *^##^	1.5 ± 0.09 *^##^

Data are the mean ± SE (n = 6). * *p* < 0.001 vs. control group; ^#^ *p* < 0.01, ^##^ *p* < 0.001 vs. TAA group (one-way ANOVA).

**Table 3:** Oleanolic acid (OA) attenuated oxidative stress parameters and boosted antioxidants in hepatic and renal tissues.

Parameters	Groups				
	Control	OA	TAA	OA 45 + TAA	OA 90 + TAA
MDA (nmol/g tissue)					
Liver	23.4 ± 1.9	20.9 ± 1.4	67.4 ± 5.2 ***	43.8 ± 2.7 ***^##^	28.5 ± 2.2 ^###^
Kidney	20.5 ± 2.5	18.7 ± 1.3	72.3 ± 5.9 ***	50.9 ± 3.7 ***^##^	33.6 ± 4.1 *^###^
4-HNE (µg/mL)					
Liver	0.27 ± 0.02	0.3 ± 0.03	1.2 ± 0.05 ***	0.8 ± 0.04 ***^##^	0.4 ± 0.03 ^###^
Kidney	0.36 ± 0.04	0.04 ± 0.04	1.3 ± 0.1 ***	0.9 ± 0.07 ***^##^	0.58 ± 0.05 *^###^
NOx (µM/L)					
Liver	35.8 ± 4.2	30.2 ± 2.8	237.2 ± 11.6 ***	120.2 ± 9.8 ***^###^	97.5 ± 4.3 **^###^
Kidney	48.9 ± 3.1	39.7 ± 3.0	256.3 ± 16.2 ***	142.1 ± 11.5 ***^###^	119.2 ± 7.2 **^###^
GSH (µmol/g tissue)					
Liver	16.7 ± 1.0	17.5 ± 1.5	5.3 ± 0.6 ***	7.8 ± 0.4 ***^#^	13.9 ± 1.2 ^###^
Kidney	15.4 ± 1.2	17.2 ± 1.3	3.2 ± 0.2 ***	8.5 ± 0.6 ***^###^	14.3 ± 0.9 ^###^
SOD (Unit/g tissue)					
Liver	26.5 ± 2.1	27.55 ± 1.4	12.4 ± 1.2 ***	18.6 ± 1.2 ***^##^	20.6 ± 1.6 ^###^
Kidney	24.2 ± 1.4	28.2 ± 1.2	11.8 ± 1.1 ***	17.2 ± 0.9 ***^##^	22.4 ± 2.1 ^###^
TAC (mmol/g tissue)					
Liver	0.94 ± 0.03	0.9 ± 0.07	0.4 ± 0.03 ***	0.55 ± 0.04 ***^#^	0.75 ± 0.05 ^###^
Kidney	0.88 ± 0.05	0.8 ± 0.04	0.3 ± 0.02 ***	0.43 ± 0.02 ***^#^	0.66 ± 0.04 ^###^

Data are the mean ± SE (n = 6). * *p* < 0.05, ** *p* < 0.01, *** *p* < 0.001 vs. control group; ^#^ *p* < 0.05, ^##^ *p* < 0.01, ^###^ *p* < 0.001 vs. TAA group (one-way ANOVA).

## Data Availability

Data is contained within the article and the Supplementary Materials.

## Supplementary Materials

The following supporting information can be downloaded at: https://www.mdpi.com/article/10.3390/medicina59071351/s1, Figure S1: ^1^H NMR spectrum of oleanolic acid (CDC_l3_, 850 MHz); Figure S2: Expansion of ^1^H NMR spectrum of oleanolic acid (CDCl_3_, 850 MHz); Figure S3: ^13^C NMR spectrum of oleanolic acid (CDCl_3_, 214 MHz); Figure S4: DEPT ^13^C NMR spectrum of oleanolic acid (CDCl_3_, 214 MHz); Figure S5: HPLC chromatogram of OA.; Table S1: NMR spectral data of OA.
