# Supplementary material for: SIRT1/Nrf2/NF-κB Signaling Mediates Anti-Inflammatory and Anti-Apoptotic Activities of Oleanolic Acid in a Mouse Model of Acute Hepatorenal Damage
Source: Medicina (Kaunas). 2023 Jul 24;59(7):1351. doi: 10.3390/medicina59071351 (PMC10383078; doi:10.3390/medicina59071351)
Supplement: Supplementary file 1 [file medicina-59-01351-s001.zip › medicina-2463144-supplementary.pdf]

Supporting Material

SIRT1/Nrf2/NF-κB Signaling Mediates Anti-Inflammatory and Anti-Apoptotic Activities of Oleanolic Acid in a Mouse Model of Acute Hepatorenal Damage

Manea A. I. Alqrad [1], Dina S. El-Agamy [2], Sabrin R. M. Ibrahim [3,4], Alaa Sirwi [1], Hossam M. Abdallah [1], Essam Abdel-Sattar [5], Ali M. El-Halawany [5], Wael M. Elsaed [6] and Gamal A. Mohamed [1,*]

1 Department of Natural Products and Alternative Medicine, Faculty of Pharmacy, King Abdulaziz University, Jeddah 21589, Saudi Arabia; mibrahimalqrad@stu.kau.edu.sa (M.A.I.A.); asirwi@kau.edu.sa (A.S.); hmafifi@kau.edu.sa (H.M.A.)

2 Department of Pharmacology and Toxicology, Faculty of Pharmacy, Mansoura University, Mansoura 35516, Egypt; dinaagamy@mans.edu.eg

3 Department of Chemistry, Preparatory Year Program, Batterjee Medical College, Jeddah 21442, Saudi Arabia; sabrin.ibrahim@bmc.edu.sa

4 Department of Pharmacognosy, Faculty of Pharmacy, Assiut University, Assiut 71526, Egypt

5 Department of Pharmacognosy, Faculty of Pharmacy, Cairo University, Giza 12613, Egypt; essam.abdelsattar@pharma.cu.edu.eg (E.A.-S.); ali.elhalawany@pharma.cu.edu.eg (A.M.E.-H.)

6 Department of Anatomy and Embryology, Faculty of Medicine, Mansoura University, Mansoura 35516, Egypt; wzaarina@mans.edu.eg

* Correspondence: gahussein@kau.edu.sa; Tel.: +966-59-763-6182

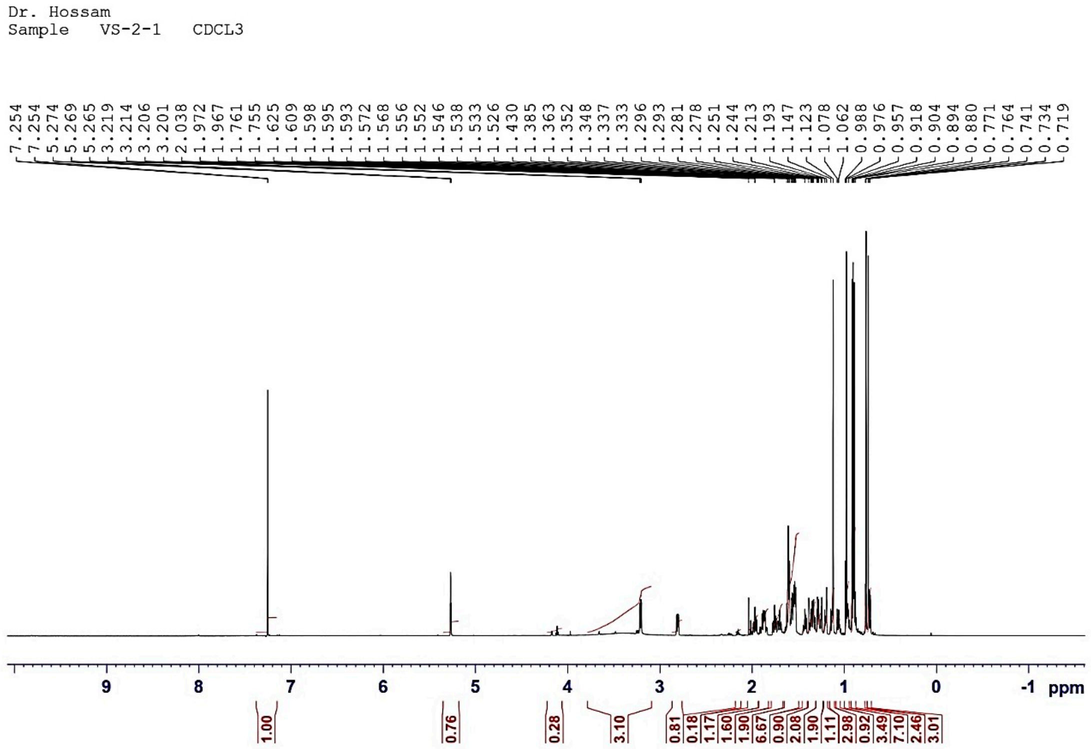

Figure S1: ^{1}H NMR spectrum of oleanolic acid ($CDCl_3$, 850 MHz).

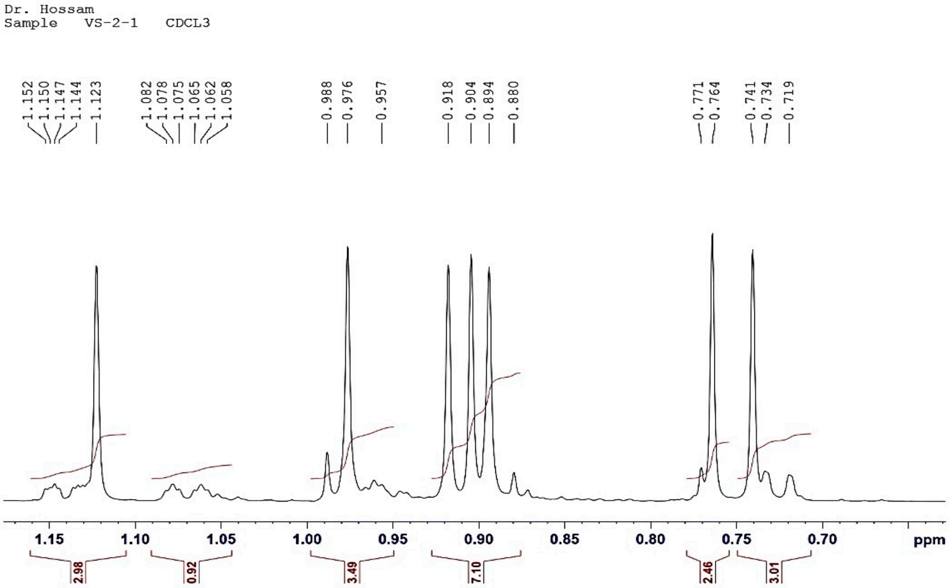

Figure S2: Expansion of ^{1}H NMR spectrum of oleanolic acid ($CDCl_3$, 850 MHz).

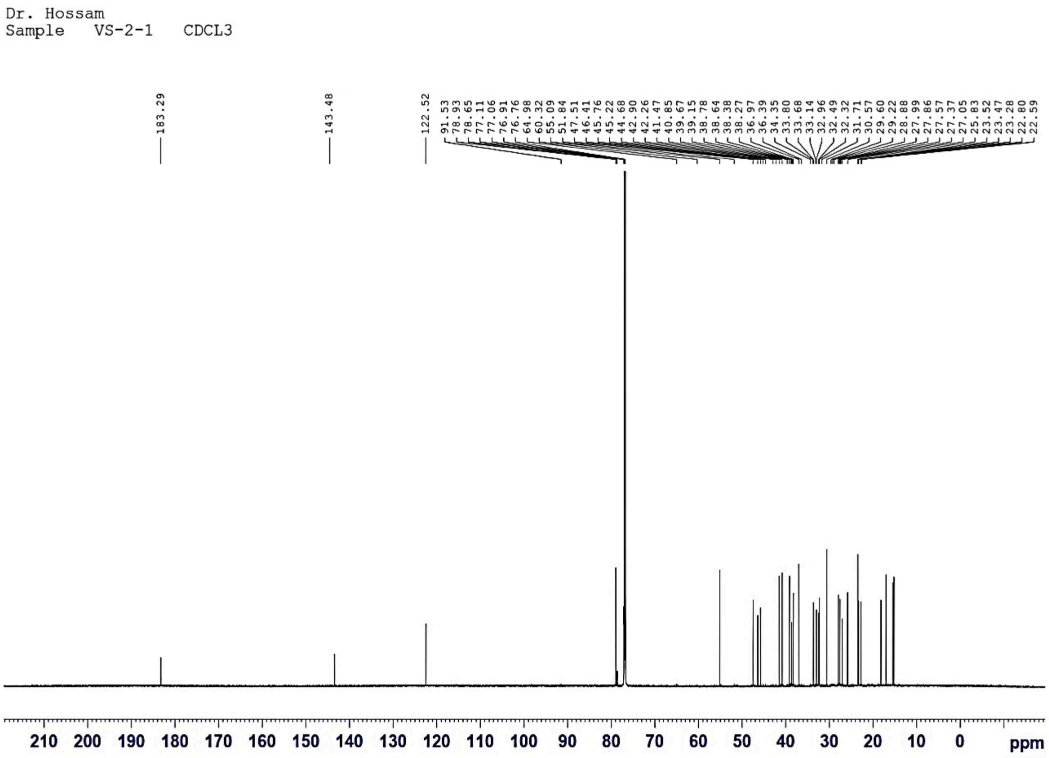

Figure S3: ^{13}C NMR spectrum of oleanolic acid ($CDCl_3$, 214 MHz).

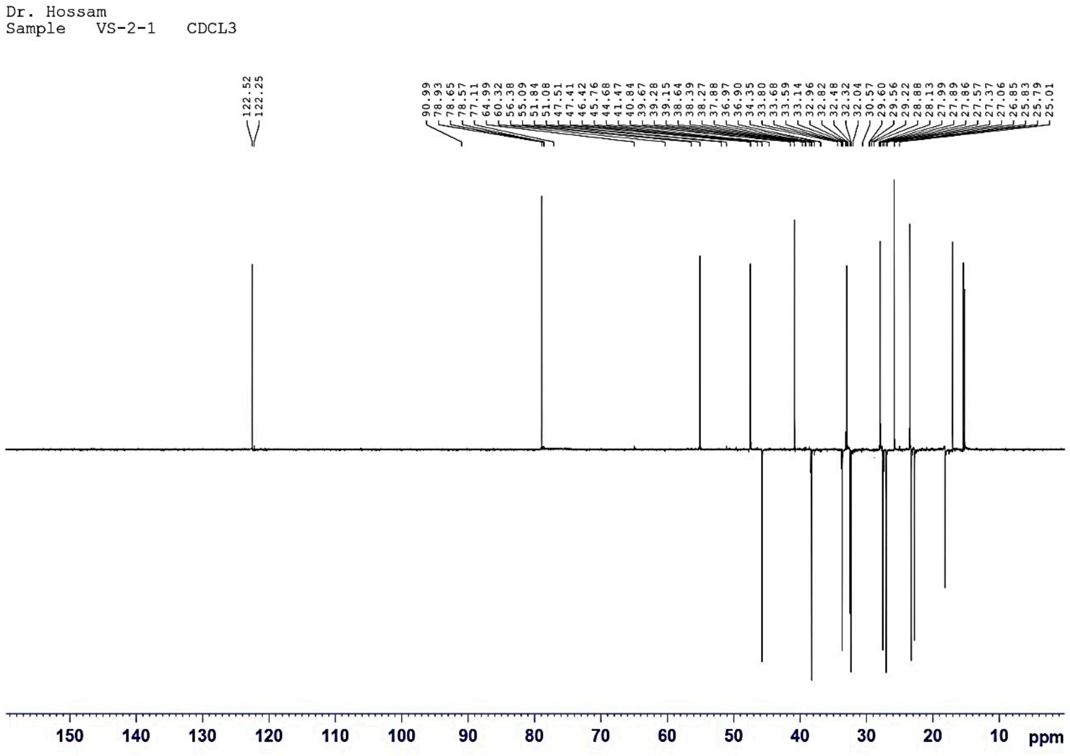

Figure S4: DEPT ^{13}C NMR spectrum of oleanolic acid ($CDCl_3$, 214 MHz).

Table S1. NMR spectral data of oleanolic acid ($CDCl_3$, 850 and 214 MHz).

No.	δ_H (m, *J* in Hz)	δ_C
**1**	1.52 m<br>1.08 m	39.2 CH_2
**2**	172 m<br>1.54 m	27.1 CH_2
**3**	3.23 dd (11.2, 4.8)	78.9 CH
**4**	-	38.2 C
**5**	0.72 m	55.1 CH
**6**	1.42 m<br>1.27 m	18.2 CH_2
**7**	1.48 m<br>1.32 m	32.5 CH_2
**8**	-	38.6 C
**9**	1.53 m	47.5 CH
**10**	-	37.0 C
**11**	1.91 m<br>1.86 m	23.3 CH_2
**12**	5.27 t (3.4)	122.5 CH
**13**	-	143.4 C
**14**	-	40.9 C
**15**	1.78 m<br>1.20 m	27.6 CH_2
**16**	1.98 m<br>1.62 m	22.6 CH_2
**17**	-	46.4 C
**18**	2.81 dd (13.6, 4.3)	41.5 CH
**19**	1.60 m<br>1.15 m	45.8 CH_2
**20**	-	30.6 C
**21**	1.42 m<br>1.28 m	34.4 CH_2
**22**	1.79 m<br>1.58 m	32.3 CH_2
**23**	0.98 s	28.0 CH_3
**24**	0.76 s	15.4 CH_3
**25**	0.89 s	15.2 CH_3
**26**	0.74 s	17.0 CH_3
**27**	1.12 s	25.8 CH_3
**28**	-	183.3 C
**29**	0.90 s	33.0 CH_3
**30**	0.92 s	23.4 CH_3

)ata File C:\CHEM32\1\DATA\SAMEH20222023\NOHA000488.D
;ample Namê: Sample Dr Gamal

===

Acq. Operator	: SAMEH-NOHA		
Acq. Instrument	: Instrument 1	Location : Vial 93	
Injection Date	: 5/21/2023 3:09:25 PM		
		Inj Volume : 1.0 µl	
Acq. Method	: C:\CHEM32\1\METHODS\SAMEH-2022-23.M		
Last changed	: 5/21/2023 3:07:54 PM by SAMEH-NOHA		
	(modified after loading)		
Analysis Method	: C:\CHEM32\1\METHODS\SAMEH-2022-23.M		
Last changed	: 5/21/2023 3:18:17 PM by SAMEH-NOHA		
	(modified after loading)		
Sample Info	: 50 % ACN		
	1 ML/Min		
	Zorbax 250x4.6 mm		

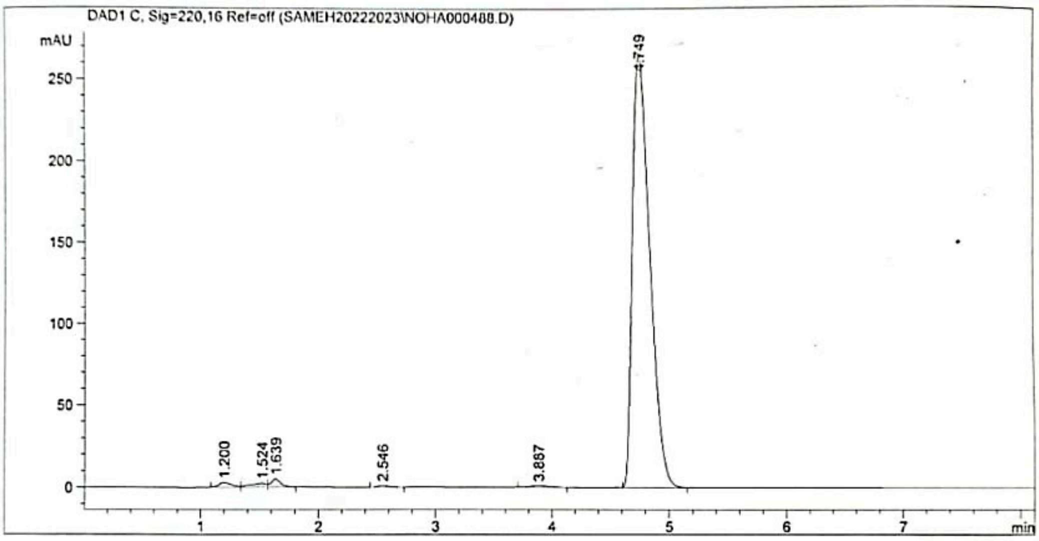

===

Area Percent Report

===

Sorted By : Signal
Multiplier: : 1.0000
Dilution: : 1.0000
Use Multiplier & Dilution Factor with ISTDs

Signal 1: DAD1 C, Sig=220,16 Ref=off

Peak #	RetTime [min]	Type	Width [min]	Area [mAU*s]	Height [mAU]	Area %
1	1.200	BV	0.1152	21.80693	2.91725	0.7729
2	1.524	VV	0.1238	21.22238	2.33968	0.7522
3	1.639	VB	0.0832	27.66283	4.94669	0.9805

Scanned with CamScanner

Data File C:\CHEM32\1\DATA\SAMEH20222023\NOHA000488.D
Sample Name: Sample Dr Gamal

Peak #	RetTime [min]	Type	Width [min]	Area [mAU*s]	Height [mAU]	Area %
4	2.546	BB	0.0950	6.55862	1.04888	0.2325
5	3.887	BB	0.1592	11.08350	1.03271	0.3929
6	4.749	BB	0.1621	2732.97021	265.38626	96.8690

Totals : 2821.30448 277.67147

*** End of Report ***

Scanned with CamScanner

Figure S5: HPLC chromatogram of oleanolic acid.
